# THE EVOLUTION OF THE PERSONAL REMINDER INFORMATION AND SOCIAL MANAGEMENT (PRISM) SYSTEM

**DOI:** 10.1093/geroni/igae098.0040

**Published:** 2024-12-31

**Authors:** Walter Boot, Neil Charness, Sara Czaja, Wendy Rogers

**Affiliations:** Weill Cornell Medicine, New York, New York, United States; Florida State University, Tallahassee, Florida, United States; Weill Cornell Medicine/Center on Aging and Behavioral Research, New York, New York, United States; University of Illinois Urbana-Champaign, Champaign, Illinois, United States

## Abstract

The development of the Personal Reminder Information and Social Management (PRISM) System began fifteen years ago with a user-centered design process that resulted in PRISM 1.0, a desktop application that ran on a personal computer and included a keyboard, mouse, and printer. However, as the technological landscape changed, so did PRISM. Following the successful implementation of PRISM 1.0, PRISM 2.0 adapted the platform to be mobile and run on a tablet. Features such as videoconferencing and multiplayer games were added based on feedback from PRISM 1.0 trial participants and the availability of new applications. To broaden the reach of PRISM 2.0, it was also translated into Spanish. Building off the successful implementation of PRISM 2.0, versions of PRISM have been adapted to serve older adults experiencing cognitive impairment. Adapted versions of PRISM, with new features, are also currently being deployed in Germany to examine their impact in two different cities. This talk will focus on how PRISM has evolved over time and has been adapted to serve different, diverse populations of older people. Throughout this talk, we will emphasize how a user-centered design process, involving older adults in diverse contexts, has served the design and deployment of PRISM, and how what has been learned from each iteration of PRISM has informed the next to maximize the usefulness and usability of the system. How advances in virtual and augmented reality, artificial intelligence, and wearables might shape future iterations of PRISM will be discussed.

